# Association of Glucagon-Like Peptide-1 Receptor Agonist vs Dipeptidyl Peptidase-4 Inhibitor Use With Mortality Among Patients With Type 2 Diabetes and Advanced Chronic Kidney Disease

**DOI:** 10.1001/jamanetworkopen.2022.1169

**Published:** 2022-03-07

**Authors:** Jia-Jin Chen, Chao-Yi Wu, Chang-Chyi Jenq, Tao-Han Lee, Chung-Ying Tsai, Hui-Tzu Tu, Yu-Tung Huang, Chieh-Li Yen, Tzung-Hai Yen, Yung-Chang Chen, Ya-Chung Tian, Chih-Wei Yang, Huang-Yu Yang

**Affiliations:** 1 Kidney Research Institute, Department of Nephrology, Chang Gung Memorial Hospital in Linkou, Chang Gung University College of Medicine, Taoyuan, Taiwan; 2 Division of Allergy, Asthma, and Rheumatology, Department of Pediatrics, Chang Gung Memorial Hospital, Chang Gung University College of Medicine, Taoyuan City, Taiwan; 3 Department of Nephrology, New Taipei Municipal TuCheng Hospital, New Taipei City, Taiwan; 4 Center for Big Data Analytics and Statistics, Chang Gung Memorial Hospital, Linkou, Taiwan; 5 Department of Health Policy and Management, Johns Hopkins Bloomberg School of Public Health, Baltimore, Maryland

## Abstract

**Importance:**

Glucagon-like peptide-1 (GLP-1) receptor agonist use is associated with reduced mortality and improved cardiovascular outcomes in the general population with diabetes. Dipeptidyl peptidase-4 (DPP-4) inhibitors are commonly used antidiabetic agents for patients with advanced-stage chronic kidney disease (CKD). The association of these 2 drug classes with outcomes among patients with diabetes and advanced-stage CKD or end-stage kidney disease (ESKD) is not well understood.

**Objective:**

To assess whether use of GLP-1 receptor agonists in a population with diabetes and advanced-stage CKD or ESKD is associated with better outcomes compared with use of DPP-4 inhibitors.

**Design, Setting, and Participants:**

This retrospective cohort study used data on patients with type 2 diabetes and stage 5 CKD or ESKD obtained from the National Health Insurance Research Database of Taiwan. The study was conducted between January 1, 2012, and December 31, 2018. Data were analyzed from June 2020 to July 2021.

**Exposures:**

Treatment with GLP-1 receptor agonists compared with treatment with DPP-4 inhibitors.

**Main Outcomes and Measures:**

All-cause mortality, sepsis- and infection-related mortality, and mortality related to major adverse cardiovascular and cerebrovascular events were compared between patients treated with GLP-1 receptor agonists and patients treated with DPP-4 inhibitors. Propensity score weighting was used to mitigate the imbalance among covariates between the groups.

**Results:**

Of 27 279 patients included in the study, 26 578 were in the DPP-4 inhibitor group (14 443 [54.34%] male; mean [SD] age, 65 [13] years) and 701 in the GLP-1 receptor agonist group (346 [49.36%] male; mean [SD] age, 59 [13] years). After weighting, the use of GLP-1 receptor agonists was associated with lower all-cause mortality (hazard ratio [HR], 0.79; 95% CI, 0.63-0.98) and lower sepsis- and infection-related mortality (HR, 0.61; 95% CI, 0.40-0.91). Subgroup analysis demonstrated a lower risk of mortality associated with use of GLP-1 receptor agonists compared with DDP-4 inhibitors among patients with cerebrovascular disease (HR, 0.33; 95% CI, 0.12-0.86) than among those without cerebrovascular disease (HR, 0.89; 95% CI, 0.71-1.12) (*P *=* *.04 for interaction).

**Conclusions and Relevance:**

Treatment with GLP-1 receptor agonists was associated with lower all-cause mortality among patients with type 2 diabetes, advanced-stage CKD, and ESKD than was treatment with DPP-4 inhibitors. Additional well-designed, prospective studies are needed to confirm the potential benefit of GLP-1 receptor agonist treatment for patients with advanced CKD or ESKD.

## Key Points

**Question:**

Is use of glucagon-like peptide-1 (GLP-1) receptor agonists among patients with diabetes and advanced-stage chronic kidney disease or end-stage kidney disease associated with better outcomes than use of dipeptidyl peptidase-4 (DPP-4) inhibitors?

**Findings:**

This cohort study comprising 27 279 participants with type 2 diabetes and advanced-stage chronic kidney disease or end-stage kidney disease revealed that the use of GLP-1 receptor agonists was associated with lower all-cause mortality and lower sepsis- and infection-related mortality than was use of DPP-4 inhibitors.

**Meaning:**

In this study, use of GLP-1 receptor agonists was associated with better outcomes compared with use of DPP-4 inhibitors among patients with type 2 diabetes and advanced-stage chronic kidney disease.

## Teaser

This cohort study assesses whether treatment with glucagon-like peptide-1 (GLP-1) receptor agonists is associated with better outcomes than treatment with dipeptidyl peptidase-4 (DPP-4) inhibitors among patients with type 2 diabetes and advanced-stage chronic kidney disease (CKD) or end-stage kidney disease.

## Introduction

Chronic kidney disease (CKD) and end-stage kidney disease (ESKD) are contributors to the health burden and are associated with increased mortality and cardiovascular events.^1,2^ Type 2 diabetes is the most common cause of CKD, and both diabetes and CKD are associated with increased all-cause mortality and increased rates of infection and cardiovascular events.^3,4^ The high mortality among patients with diabetes and CKD or ESKD is mostly attributable to cardiovascular or infection-related events.^5,6^ Both glucagon-like peptide-1 (GLP-1) receptor agonists and sodium-glucose cotransporter-2 (SGLT-2) inhibitors are associated with better blood glucose control, greater body weight reduction, lower mortality, and lower incidence of cardiovascular events in the general population with diabetes.^7,8,9^ According to the American Diabetes Association guideline,^10^ GLP-1 receptor agonist treatment is recommended for patients with diabetes and CKD who have an estimated glomerular filtration rate less than 60 mL/min/1.73 m^2^ and are at risk for cardiovascular disease. According to the Kidney Disease: Improving Global Outcomes 2020 Clinical Practice Guideline for Diabetes Management in Chronic Kidney Disease,^11,12^ GLP-1 receptor agonist treatment is suggested for individuals who are unable to use metformin or SGLT-2 inhibitors. Unlike SGLT-2 inhibitors, GLP-1 receptor agonists can be used in patients with advanced-stage CKD or ESKD. A recent network meta-analysis^7^ demonstrated potentially different clinical outcomes after use of GLP-1 receptor agonists and use of dipeptidyl peptidase-4 (DPP-4) inhibitors in the general population with diabetes. In that study, use of GLP-1 receptor agonists was associated with improved survival compared with use of DPP-4 inhibitors.

However, randomized clinical trials have usually excluded or enrolled small numbers of patients with advanced-stage CKD or ESKD.^13,14^ Meanwhile, DPP-4 inhibitors are commonly prescribed to patients with advanced-stage CKD or ESKD owing to their safety profiles and effectiveness for glucose control^15^ despite the neutral effect on the kidneys and cardiovascular outcomes.^15^ However, the association of GLP-1 receptor agonist treatment with improved survival has been discussed less frequently for patients with advanced CKD or ESKD. Furthermore, to our knowledge, there have been no real-world studies comparing GLP-1 receptor agonists with DPP-4 inhibitors for treatment of patients with advanced-stage CKD or ESKD. In this study, we aimed to assess whether use of GLP-1 receptor agonists among patients with type 2 diabetes and stage 5 CKD or ESKD was associated with better outcomes compared with use of DPP-4 inhibitors.

## Methods

### Data Source

This retrospective cohort study was built on data from the National Health Insurance Research Database (NHIRD) in Taiwan. Taiwan’s national health insurance system is a mandatory national health insurance program that covers more than 99.9% of Taiwan’s population. The NHIRD data sets contain deidentified information such as age, sex, examinations, medications, interventions, surgeries, admissions, and outpatient clinics. Diseases and outcomes were identified in the NHIRD using the *International Classification of Diseases, Ninth Revision, Clinical Modification* (*ICD-9-CM*), and the *International Statistical Classification of Diseases and Related Health Problems, Tenth Revision* (*ICD-10*). Detailed information regarding the national health insurance system as a reliable source for real-world studies has been demonstrated previously.^16,17,18^ This study was approved by the institutional review board of the Chang Gung Memorial Hospital. The need for individual informed consent was waived by the institutional review board of Chang Gung Memorial Hospital because personal identification data were not included in the NHIRD. This study followed the Strengthening the Reporting of Observational Studies in Epidemiology (STROBE) reporting guideline and the RECORD-PE guideline.^19^

### Study Population

This study included data for patients with type 2 diabetes and stage 5 CKD or ESKD obtained from January 1, 2012, to December 31, 2018. Stage 5 CKD was identified using a combination of *ICD-9-CM* (016.0, 042, 095.4, 189, 223, 236.9, 250.4, 271.4, 274.1, 403-404, 440.1, 442.1, 446.21, 447.3, 572.4, 580-589, 590-591, 593, 642.1, 646.2, 753, and 984) and *ICD-10* (B20, A52.75, C64-68, D30, D41, E11.29, E74.8, M10.30, N20, I70.1, I72.2, M31, I77.3, K76.7, N00-08, N10-15, N18, N28.83, N28.81, N28.1, N28.9, O10.41-43, O12.14, O26.831-839, and Q60-4) codes and a prescription for erythropoiesis-stimulating agents. End-stage kidney disease requiring dialysis was verified by identifying insured individuals who had a diagnostic code for ESKD (either *ICD-9-CM* code 585 or *ICD-10* code N186) and received an appropriate catastrophic illness certificate for maintenance dialysis. Patients who were first prescribed either a GLP-1 receptor agonist or a DPP-4 inhibitor 3 months before the index date were included. The index date was defined as 91 days after the first exposure to a GLP-1 receptor agonist or DPP-4 inhibitor. We excluded patients who had incomplete data, were younger than 20 years, had major adverse cardiovascular and cerebrovascular events (MACCE) 90 days before the index date, had a malignant neoplasm detected, or underwent a kidney transplant or dialysis or died 90 days before the index date (Figure). We divided the eligible patients into 2 groups based on the use of GLP-1 receptor agonists or DPP-4 inhibitors during the 3-month exposure window.

**Figure.**
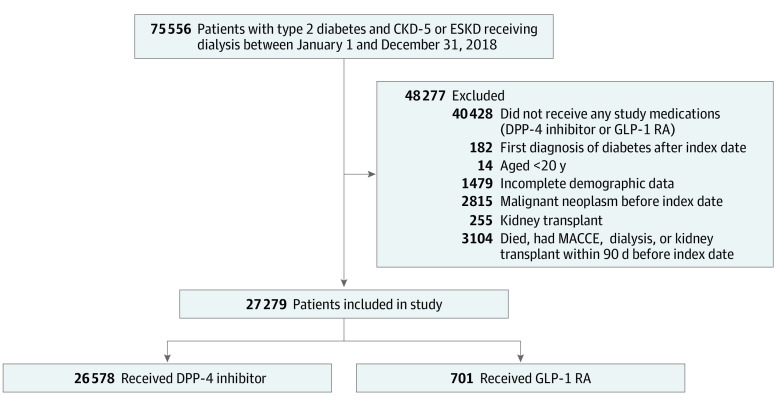
Flowchart of Patient Enrollment CKD-5 indicates stage 5 chronic kidney disease; DPP-4 inhibitor, dipeptidyl peptidase 4 inhibitor; ESKD, end-stage kidney disease; GLP-1 RA, glucagon-like peptide-1 receptor agonist; MACCE, major adverse cardiovascular or cerebrovascular event.

### Covariates

Age, sex, area of residence (urban or rural area), income level, occupation, and 10 comorbidities (hypertension, dyslipidemia, cirrhosis, systemic lupus erythematosus, atrial fibrillation, peripheral arterial disease, coronary artery disease or ischemic heart disease, heart failure, cerebrovascular disease, and ESKD requiring dialysis) were used as covariates (eTable 1 in the Supplement). Overall health was measured using the Charlson Comorbidity Index.^20^ The baseline characteristics of the patients were identified before the index date to track any history of major comorbidities from the NHIRD. Comorbidities were defined as at least 1 inpatient diagnosis or 2 outpatient diagnoses of a given disease within 1 year before the index date. Medications were also evaluated by review of prescription records in the NHIRD. The following medications listed in prescription records within 90 days before the index date were extracted: angiotensin-converting enzyme inhibitors, angiotensin receptor blockers, β-blockers, calcium channel blockers, aspirin, diuretics, fibrate, gemfibrozil, nonsteroidal anti-inflammatory drugs, or other types of antidiabetic medications (sulfonylurea, α-glucosidase inhibitors, insulin, and meglitinide analogs).

### Outcome Measurement

The primary outcome was all-cause mortality after the index date during the follow-up period. The secondary outcomes were sepsis- or infection-related mortality and MACCE-related mortality. MACCE included myocardial infarction, cardiogenic shock, heart failure, coronary revascularization, coronary artery bypass surgery, thrombolysis therapy, malignant arrhythmia, and stroke (eTable 1 in the Supplement). MACCE-related mortality was identified by the discharge death code (*ICD-9-CM*: 390-392, 393-398, 410-414, 420-429, 430-438, and 440; *ICD-10*: I10-15, I01-02, I05-09, I20-25, I27, I30-52, I60-69, and I70-71) or in-hospital mortality with a diagnostic code for a MACCE^21,22^ (eTable 1 in the Supplement). Infection- and sepsis-related mortality was also identified using the discharge death code (*ICD-9-CM*: 010-016, 033, 036-038, 320-322, 480-487, and 490-493; *ICD-10*: A15-19, A40-41, G00, G03, and J10-J18) or in-hospital mortality with the diagnostic code for infection or sepsis (eTable 1 in the Supplement). All patients were monitored until death or December 31, 2018. For survival analysis, the follow-up period was 4 years.

### Statistical Analysis

To reduce confounding and selection bias, the propensity score weighting method was used to balance covariates between the 2 groups; these included baseline characteristics, comorbidities, antidiabetic agents, antihypertensive agents, ESKD requiring dialysis, and Charlson Comorbidity Index score. The absolute standardized mean difference was used as a metric of propensity score performance for both GLP-1 receptor agonists and DPP-4 inhibitors.^23^ Absolute standardized mean difference values that were higher than 0.1 indicated an imbalance between groups.^23^ The incidence of the outcome of interest was computed as the total study outcomes during the follow-up period divided by person-years at risk. The hazard ratio (HR) with 95% CI of each outcome was estimated using a Cox proportional hazards regression model. The event-free survival rate among patients treated with GLP-1 receptor agonists vs DPP-4 inhibitors was evaluated using the Kaplan-Meier method, and a *P* value of survival analysis was calculated by log-rank test. For the survival analysis, follow-up duration was estimated from the index date to first outcome event diagnosis (all-cause mortality, MACCE-related mortality, or sepsis-related mortality, defined by the date of health insurance policy termination) or until December 31, 2018, for uncensored participants and the end of the 4-year follow-up period for those who were censored. For MACCE-related mortality and sepsis-related mortality, participants with health insurance policy termination (mostly because of death) owing to other causes of death were censored.

Subgroup analysis was conducted to explore potential different treatment effects between subgroups by age (<65 years or ≥65 years), sex, diagnosis of coronary artery disease or ischemic heart disease, diagnosis of cerebrovascular disease, and use of an angiotensin-converting enzyme inhibitor or angiotensin receptor blocker and ESKD requiring dialysis. In subgroup analysis, the Cox proportional hazards regression model and subgroup-specific HRs were used to estimate subgroup-specific mean treatment effect. Comparison between subgroups was performed after independent inverse probability of treatment weighting adjustment for covariates (atrial fibrillation, congestive heart failure, hypertension, cirrhosis, peripheral arterial disease, systemic lupus erythematosus, dyslipidemia, and use of prescription drugs) and unselected subgroup specific covariates. Results of subgroup analysis were shown using a forest plot (eFigure 2 in the Supplement). Sensitivity analyses were also conducted to exclude (1) participants with a history of switching between GLP-1 receptor agonists and DPP-4 inhibitors and (2) those who were outliers in the propensity score–matched analysis. Data were analyzed from June 2020 to July 2021. Statistical significance was defined at 2-sided *P* < .05. All statistical analyses were conducted using SAS, version 9.4 (SAS Institute).

## Results

### Patient Characteristics

A total of 75 556 patients with type 2 diabetes and stage 5 CKD or ESKD who were receiving dialysis were identified between January 1, 2012, and December 31, 2018. A total of 48 277 patients were excluded because they did not use target medications (n = 40 428) or fulfilled the exclusion criteria (incomplete data [n = 1479], diagnosis of diabetes after enrollment [n = 182], <20 years of age [n = 14], malignant neoplasm [n = 2815]; kidney transplant [n = 255]; and diagnosis of MACCE, receipt of dialysis, or death before the index date [n = 3104]) (Figure). Of 27 279 patients included in the study, 26 578 were in the DPP-4 inhibitor group (14 443 [54.34%] male; mean [SD] age, 65 [13] years) and 701 in the GLP-1 receptor agonist group (346 [49.36%] male; mean [SD] age, 59 [13] years). Baseline demographic characteristics, comorbidities, and medications prescribed for the 2 groups are summarized in Table 1. Before propensity score weighting, the DPP-4 inhibitor group was older, concentrated in rural areas, and included fewer patients receiving dialysis and more patients receiving an angiotensin-converting enzyme inhibitor, diuretics, and insulin compared with the GLP-1 receptor agonist group. After propensity score weighting, all analyzed covariates were balanced between the 2 groups. After propensity score weighting, the DPP-4 inhibitor group consisted of 26 568 patients, and the GLP-1 receptor agonist group consisted of 603 patients. The mean (SD) age was 66 (13) years in the DPP-4 inhibitor group and 65 (11) years in the GLP-1 receptor agonist group. In the DPP-4 inhibitor group, 14 414 patients (54.25%) were male, and in the GLP-1 receptor agonist group, 319 (52.90%)were male. Hypertension was the most common comorbidity in the enrolled population; after propensity score weighting, 22 369 patients (84.20%) in the DPP-4 inhibitor group and 506 (83.92%) in the GLP-1 receptor agonist group had hypertension. The mirrored histogram of propensity score distribution showed substantial overlap of propensity scores between the 2 groups. For primary and second outcomes, the median follow-up duration was 3.57 years (IQR, 1.95-4.00 years) in the DPP-4 inhibitor group and 1.76 years (IQR, 1.03-3.89 years) in the GLP-1 receptor agonist group.

**Table 1.** Baseline Characteristics of Patients With Diabetes and Stage 5 Chronic Kidney Disease or End-Stage Kidney Disease

Characteristic	Before propensity score weighting			After propensity score weighting		
	Patients, No. (%)		ASMD	Patients, No. (%)		ASMD
	DPP-4 inhibitor (n = 26 578)	GLP-1 receptor agonist (n = 701)		DPP-4 inhibitor (n = 26 568)	GLP-1 receptor agonist (n = 603)	
Age, mean (SD)	65 (13)	59 (13)	0.470	66 (13)	65 (11)	0.029
Sex						
Female	12 135 (46.66)	355 (50.64)	0.100	12 154 (45.75)	284 (47.10)	0.027
Male	14 443 (54.34)	346 (49.36)		14 414 (54.25)	319 (52.90)	
Area of residence						
Urban	6584 (24.77)	260 (29.39)	0.140	6605 (24.86)	155 (25.70)	0.028
Suburban	7440 (27.99)	219 (31.24)		7455 (28.06)	161 (26.67)	
Rural	12 554 (47.23)	276 (39.37)		12 509 (47.08)	287 (47.64)	
Occupation						
Dependent	10 276 (38.66)	242 (34.52)	0.210	10 244 (38.56)	232 (38.42)	0.038
Civil servant	282 (1.06)	11 (1.57)		284 (1.07)	7 (1.15)	
Nonmanual worker	2109 (7.94)	100 (14.27)		2146 (8.08)	55 (9.09)	
Manual worker	8932 (33.61)	222 (31.67)		8921 (33.58)	204 (33.86)	
Other	4979 (18.73)	126 (17.97)		4974 (18.72)	105 (17.49)	
Comorbidities						
Hypertension	22 388 (84.24)	579 (82.60)	0.044	22 369 (84.20)	506 (83.92)	0.007
Dyslipidemia	11 990 (45.11)	348 (49.64)	0.091	12 010 (45.20)	284 (47.07)	0.038
Cirrhosis	634 (2.39)	17 (2.43)	0.003	632 (2.38)	14 (2.39)	<0.001
SLE	47 (0.18)	4 (0.57)	0.065	48 (0.18)	1 (0.17)	0.026
Atrial fibrillation	561 (2.11)	9 (1.28)	0.064	558 (2.10)	10 (1.70)	0.029
Peripheral arterial disease	1231 (4.63)	36 (5.14)	0.023	1230 (4.63)	30 (4.97)	0.016
Coronary artery disease/ischemic heart disease	5566 (20.94)	156 (22.25)	0.032	5567 (20.95)	122 (20.19)	0.019
Heart failure	4631 (17.42)	120 (17.12)	0.008	4624 (17.40)	120 (19.84)	0.063
Cerebrovascular disease	3311 (12.46)	76 (10.84)	0.050	3302 (12.43)	68 (11.29)	0.035
Dialysis	5819 (21.89)	208 (29.67)	0.180	5862 (22.06)	136 (22.53)	0.011
Charlson Comorbidity Index score, mean (SD)	2.98 (1.49)	2.90 (1.41)	0.059	2.98 (1.49)	2.90 (1.31)	0.062
Medications						
ACEI	2864 (10.78)	41 (5.85)	0.180	2835 (10.67)	56 (9.24)	0.048
ARB	16 216 (61.01)	449 (64.05)	0.063	16 220 (61.05)	380 (62.93)	0.039
Aspirin	7898 (29.72)	246 (35.09)	0.120	7922 (29.82)	190 (31.54)	0.037
β-Blocker	8745 (32.90)	261 (37.23)	0.091	8760 (32.97)	196 (32.47)	0.011
CCB	18 999 (71.48)	482 (68.76)	0.060	18 977 (71.43)	423 (70.05)	0.030
Diuretic	14 826 (55.78)	340 (48.50)	0.150	14 783 (55.64)	320 (53.09)	0.051
Fibrate	1910 (7.19)	90 (12.84)	0.190	1941 (7.31)	44 (7.33)	<0.001
Gemfibrozil	572 (2.15)	21 (3.00)	0.053	577 (2.17)	11 (1.80)	0.026
NSAID	8381 (31.53)	207 (29.53)	0.044	8371 (31.51)	179 (29.60)	0.042
Sulfonylurea	12 004 (45.17)	311 (44.37)	0.016	11 999 (45.16)	254 (42.14)	0.061
Acarbose	3730 (14.03)	109 (15.55)	0.043	3730 (14.04)	84 (13.98)	0.002
Insulin	7813 (29.40)	114 (16.26)	0.320	7732 (29.10)	164 (27.13)	0.044
Meglitinide	6911 (26.00)	168 (23.97)	0.047	6899 (25.97)	143 (23.71)	0.052

Abbreviations: ACEI, angiotensin-converting enzyme inhibitor; ARB, angiotensin receptor blocker; ASMD, absolute standardized mean difference; CCB, calcium channel blocker; DPP-4, dipeptidyl peptidase-4; GLP-1, glucagon-like peptide-1; NSAID, nonsteroidal anti-inflammatory drug; SLE, systemic lupus erythematosus.

### Primary and Secondary Outcomes

The rates of all-cause mortality, sepsis- or infection-related mortality, and MACCE-related mortality in the 2 study groups are shown in Table 2. The rate of all-cause mortality was 7.95 per 100 person-years (95% CI, 7.76-8.15 per 100 person-years) in the DPP-4 inhibitor group and 6.10 per 100 person-years (95% CI, 4.76-7.45 per 100 person-years) in the GLP-1 receptor agonist group. After propensity score weighting, use of GLP-1 receptor agonists was associated with lower all-cause mortality compared with use of DPP-4 inhibitors (HR, 0.79; 95% CI, 0.63-0.98; *P* = .03). The rate of sepsis- or infection-related mortality was 3.01 per 100 person-years (95% CI, 2.88-3.13 per 100 person-years) in the DPP-4 inhibitor group and 1.80 per 100 person-years (95% CI, 1.07-2.53 per 100 person-years) in the GLP-1 receptor agonist group. There was a lower risk for sepsis- or infection-related mortality in the GLP-1 receptor agonist group vs the DPP-4 inhibitor group was (HR, 0.61; 95% CI, 0.40-0.91; *P* = .02). The rate of MACCE-related mortality was 2.56 per 100 person-years (95% CI, 2.45-2.67 per 100 person-years) in the DPP-4 inhibitor group and 2.64 per 100 person-years (95% CI, 1.75-3.53 per 100 person-years) in the GLP-1 receptor agonist group. MACCE-related mortality in the GLP-1 receptor agonist group was similar to that in the DPP-4 inhibitor group (HR, 1.07; 95% CI, 0.76-1.51; *P* = .69) (Table 2). The survival curve analysis for all-cause mortality, sepsis- and infection-related mortality, and MACCE-related mortality after propensity score weighting is provided in eFigure 1 in the Supplement.

**Table 2.** Analysis of Primary and Secondary Outcomes

Event type	DPP-4 inhibitors		GLP-1 receptor agonists		GLP-1 receptor agonists vs DPP-4 inhibitors	
	Events, No.^a^	Rate, per 100 person-years (95% CI)	Events, No.^b^	Rate, per 100 person-years (95% CI)	Hazard ratio (95% CI)	*P* value
Before propensity score weighting						
MACCE-related mortality	2009	2.57 (2.46-2.68)	39	2.60 (1.78-3.42)	1.05 (0.77-1.44)	.76
Sepsis- and infection-related mortality	2366	3.03 (2.91-3.15)	24	1.60 (0.96-2.24)	0.54 (0.36-0.80)	.003
All-cause mortality	6252	8.00 (7.80-8.20)	81	5.40 (4.23-6.58)	0.69 (0.56-0.86)	.001
After propensity score weighting						
MACCE-related mortality	1999	2.56 (2.45-2.67)	34	2.64 (1.75-3.53)	1.07 (0.76-1.51)	.69
Sepsis- and infection-related mortality	2347	3.01 (2.88-3.13)	23	1.80 (1.07-2.53)	0.61 (0.40-0.91)	.02
All-cause mortality	6212	7.95 (7.76-8.15)	79	6.10 (4.76-7.45)	0.79 (0.63-0.98)	.03

Abbreviations: DPP-4, dipeptidyl peptidase-4; GLP-1, glucagon-like peptide-1; MACCE, major cardiac and cerebrovascular event.

^a^ There were 78 127.49 person-years at risk before propensity score weighting and 78 096.63 person-years at risk after propensity score weighting.

^b^ There were 1499.55 person-years at risk before propensity score weighting and 1289.57 person-years at risk after propensity score weighting.

### Subgroup Analysis and Sensitivity Analysis

eFigure 2 in the Supplement presents the results of the subgroup analysis. A difference in all-cause mortality was identified between the use of GLP-1 receptor agonists and use of DDP-4 inhibitors among participants with and without cerebrovascular disease. Among participants with cerebrovascular disease, there was lower risk for all-cause mortality in the GLP-1 receptor group than in the DDP-4 inhibitor group (HR, 0.33; 95% CI, 0.12-0.86). This association was not observed among individuals without cerebrovascular disease (HR, 0.89; 95% CI, 0.71-1.12) (*P *=* *.04 for interaction). For MACCE-related mortality and for sepsis- and infection-related mortality, no significant treatment effect heterogeneity was detected.

Some enrolled participants switched from the GLP-1 receptor agonist group to the DDP-4 inhibitor group after the first GLP-1 receptor agonist prescription. We conducted sensitivity analysis to exclude these patients with a history of drug switching, and the results are presented as eTable 2 in the Supplement. Outliers in the propensity score–weighted analysis were included in further sensitivity analysis, and the results are shown in eTables 3 and 4 in the Supplement. The aforementioned lower all-cause and sepsis-related mortality associated with GLP-1 receptor agonist treatment compared with DPP-4 inhibitor treatment did not differ in each sensitivity analysis.

## Discussion

The 2 major findings of this study can be summarized as follows: (1) use of GLP-1 receptor agonists in patients with diabetes and stage 5 CKD or EKSD was associated with lower all-cause mortality compared with use of DPP-4 inhibitors and (2) use of GLP-1 receptor agonists was associated with lower sepsis- and infection-related mortality compared with use of DPP-4 inhibitors. A previous study reported that use of GLP-1 receptor agonists was associated with lower all-cause mortality, cardiovascular-related mortality, and kidney failure compared with placebo in the general population with diabetes.^7^ In that study, use of GLP-1 receptor agonists was also associated with improved outcomes compared with several traditional antidiabetic agents, including sulfonylureas, DPP-4 inhibitors, and thiazolidinedione. Nevertheless, the proportions of participants with advanced-stage CKD in large randomized clinical trials (Liraglutide Effect and Action in Diabetes: Evaluation of Cardiovascular Outcome Results [LEADER] trial^13^ and Researching Cardiovascular Events With a Weekly Incretin in Diabetes [REWIND] trial^14^) examining the clinical effects of GLP-1 receptor agonists were small despite enrollment of these patients in the trials. Real-world evidence regarding use of treatment with GLP-1 receptor agonists among patients with diabetes and late-stage CKD or ESKD is lacking. The lower all-cause mortality in the GLP-1 receptor agonist group compared with the DPP-4 inhibitor group among patients with diabetes and advanced-stage CKD or ESKD in our study is in agreement with the findings of Palmer et al.^7^

The difference between DPP-4 inhibitors and GLP-1 receptor agonists has been studied previously.^7,24,25,26,27,28,29,30,31,32,33,34,35,36,37,38,39,40,41,42,43^ Both drugs are based on the incretin pathway; however, DPP-4 inhibitors and GLP-1 receptor agonists showed different clinical efficacy.^25,26,27,28,29,30,31,32,33,34,35,36,37,38,39,40,41,42,43^ Lower hemoglobin A1c levels,^24,25,26,27,28,29,30,31,32,33,34^ greater body weight reduction,^25,26,27,31,32,33,35,36,37,38,39,40^ improved β-cell function,^25,26,28,33,36^ improved cardiac function,^27,41,42^ and reduced albumin level^27,42,43^ were observed in randomized clinical trials comparing GLP-1 receptor agonists with DPP-4 inhibitors. The different clinical benefits (ie, better glucose control, metabolic benefit) between GLP-1 receptor agonists and DPP-4 inhibitors might explain the better outcomes observed in patients treated with GLP-1 receptor agonists than in those treated with DPP-4 inhibitors.^7^ These differences are summarized in eTable 5 in the Supplement.

Increased susceptibility to sepsis and poor outcomes after sepsis have been well documented in patients with diabetes and CKD.^44,45^ In the present study, we observed lower sepsis- and infection-related mortality in the GLP-1 receptor agonist group than in the DPP-4 inhibitor group. Incretin-based therapy might mitigate excessive inflammation and microvascular thrombosis in sepsis via activation of the GLP-1 receptor.^46,47^ However, differences in the infection-related outcomes (including outcomes of COVID-19) between patients receiving DPP-4 inhibitor and GLP-1 receptor agonist treatment have been observed.^48,49^ Increased mortality was seen in the population of patients with diabetes and COVID-19 treated with DPP-4 inhibitors (HR, 1.07; 95% CI, 1.01-1.13).^48^ In consideration of the burden of sepsis-related mortality in populations with advanced CKD and ESKD, the findings of the present study deserve further prospective trials for confirmation.

We observed that MACCE-related mortality did not differ between the 2 groups, which was similar to the result from a study of the general population with diabetes.^7^ Similar findings were observed in patients with ESKD in whom the cardiovascular protective effect of statins was lost.^50,51,52,53^ Previously published randomized clinical trials and real-world observational studies^50,51,52,53^ revealed that use of statins for patients with ESKD or stage 5 CKD who were not receiving dialysis did not provide cardiovascular benefit. Gupta et al^54^ reported that a statin prescription was associated with a lower risk of sepsis events. Furthermore, the relatively short follow-up period and small sample size in the present study might be the reason that we did not observe lower MACCE-related mortality among patients treated with GLP-1 receptor agonists. In summary, we observed lower all-cause mortality and sepsis-related mortality but not lower MACCE-related mortality in the GLP-1 receptor agonist group compared with the DPP-4 inhibitor group. The study could not assess causal relationships, and an explanation for our finding is unclear.

### Limitations

This study has several limitations. First, this was a retrospective cohort study lacking detailed information on clinical factors (eg, glucose control) and other factors that might confound the outcome (eg, smoking, body weight control). Some important factors such as mean estimated glomerular filtration rate, mean blood pressure, lipid control level, and the degree of albuminuria in patients who did not have ESKD were also unavailable for analysis in the NHIRD data set. Second, we could not examine the dose effect, and the indication of drug prescription might have resulted in differences between the 2 study groups. The lack of a protocolized approach to DPP-4 inhibitor treatment initiation should be noted. Furthermore, the national health insurance system in Taiwan covers a GLP-1 receptor agonist prescription only when patients have known cardiovascular disease, cerebrovascular disease, or poor glucose control despite other treatment. This policy also may have resulted in potential heterogeneity between the GLP-1 receptor agonist and DPP-4 inhibitor groups. To overcome this problem, we used the propensity score weighting method with the Charlson Comorbidity Index and as many covariates as available. Third, drug adherence could not be evaluated owing to the retrospective nature of the cohort design. Fourth, potential differences in treatment effects across subgroups might have existed, but owing to the relatively small sample size, we were unable to assess the significance of these differences across subgroups. Fifth, owing to the limited number of participants, we pooled patients with stage 5 CKD who were not receiving dialysis with those who had ESKD and were receiving dialysis. Patients with ESKD and those with CKD who were not receiving dialysis are 2 different populations. Furthermore, some patients with stage 5 CKD without a prescription for an erythropoiesis-stimulating agent were not included in this study. To overcome this baseline heterogeneity, we conducted a subgroup analysis to examine whether there was a significantly different treatment effect between these 2 populations.

## Conclusions

In this cross-sectional study, in patients with type 2 diabetes and stage 5 CKD or ESKD, use of GLP-1 receptor agonists was associated with better outcomes, including all-cause mortality and sepsis- and infection-related mortality, compared with use of DPP-4 inhibitors. Additional large-scale, prospective studies are needed to examine our results.

## Notes

Supplement. **eFigure 1.** Kaplan-Meier Survival Curves of All-Cause Mortality **eFigure 2.** Forest Plot of Subgroup Analysis for Primary and Secondary Outcomes **eTable 1.** ICD-9 and ICD-10 Diagnostic Codes **eTable 2.** Sensitivity Analysis of the Primary Outcome and Secondary Outcome After Excluding Participants With Drug Switch History **eTable 3.** Sensitivity Analysis of the Primary Outcome and Secondary Outcome After Excluding Outliers Participants With Propensity Method Value Which Are Two Times Standard Deviations From Mean **eTable 4.** Sensitivity Analysis of the Primary Outcome and Secondary Outcome After Excluding Outliers Participants With Propensity Method Value Which Are Three Times Standard Deviations From Mean **eTable 5.** Randomized Controlled Trials Comparing Clinical Effect Between GLP-1 RA and DDP-4 Inhibitors With Significant Difference
