# Supplementary material for: Association of Glucagon-Like Peptide-1 Receptor Agonist vs Dipeptidyl Peptidase-4 Inhibitor Use With Mortality Among Patients With Type 2 Diabetes and Advanced Chronic Kidney Disease
Source: JAMA Netw Open. 2022 Mar 7;5(3):e221169. doi: 10.1001/jamanetworkopen.2022.1169 (PMC8902651; doi:10.1001/jamanetworkopen.2022.1169)
Supplement: Supplement. — eFigure 1. Kaplan-Meier Survival Curves of All-Cause Mortality eFigure 2. Forest Plot of Subgroup Analysis for Primary and Secondary Outcomes eTable 1. ICD-9 and ICD-10 Diagnostic Codes eTable 2. Sensitivity Analysis of the Primary Outcome and Secondary Outcome After Excluding Participants With Drug Switch History eTable 3. Sensitivity Analysis of the Primary Outcome and Secondary Outcome After Excluding Outliers Participants With Propensity Method Value Which Are Two Times Standard Deviations From Mean eTable 4. Sensitivity Analysis of the Primary Outcome and Secondary Outcome After Excluding Outliers Participants With Propensity Method Value Which Are Three Times Standard Deviations From Mean eTable 5. Randomized Controlled Trials Comparing Clinical Effect Between GLP-1 RA and DDP-4 Inhibitors With Significant Difference [file jamanetwopen-e221169-s001.pdf]

Supplementary Online Content

Chen JJ, Wu CY, Jenq CC, et al. Association of glucagon-like peptide-1 receptor agonist vs dipeptidyl peptidase-4 inhibitor use with mortality among patients with type 2 diabetes and advanced chronic kidney disease. *JAMA Netw Open*. 2022;5(3):e221169. doi:10.1001/jamanetworkopen.2022.1169

eFigure 1. Kaplan-Meier Survival Curves of All-Cause Mortality
eFigure 2. Forest Plot of Subgroup Analysis for Primary and Secondary Outcomes
eTable 1. ICD-9 and ICD-10 Diagnostic Codes
eTable 2. Sensitivity Analysis of the Primary Outcome and Secondary Outcome After Excluding Participants With Drug Switch History
eTable 3. Sensitivity Analysis of the Primary Outcome and Secondary Outcome After Excluding Outliers Participants With Propensity Method Value Which Are Two Times Standard Deviations From Mean
eTable 4. Sensitivity Analysis of the Primary Outcome and Secondary Outcome After Excluding Outliers Participants With Propensity Method Value Which Are Three Times Standard Deviations From Mean
eTable 5. Randomized Controlled Trials Comparing Clinical Effect Between GLP-1 RA and DDP-4 Inhibitors With Significant Difference

This supplementary material has been provided by the authors to give readers additional information about their work.

© 2022 Chen JJ et al. *JAMA Network Open*.

Supplementary Profile

eFigure Legends

eFigure 1. Kaplan-Meier survival curves of all-cause mortality (A), sepsis/infection-related mortality (B), MACCE-related mortality (C) during follow-up after propensity score weighting

Abbreviations: Dipeptidyl peptidase 4 inhibitor, DPP4-inhibitor; GLP-1 RA; MACCE, major adverse cardiac cerebrovascular event

eFigure 2. Forest plot of subgroup analysis for primary and secondary outcomes. (A) All-cause mortality; (B) MACCE-related mortality; (C) sepsis/infection-related mortality

Abbreviations: ACEI, angiotensin-converting enzyme inhibitor; ARB, angiotensin receptor blocker; Dipeptidyl peptidase 4 inhibitor, DPP4-inhibitor; GLP-1 RA; MACCE, major adverse cardiac cerebrovascular event

© 2022 Chen JJ et al. *JAMA Network Open*.

eFigure 1A. Kaplan-Meier survival curves of all-cause mortality during follow-up after propensity score weighting

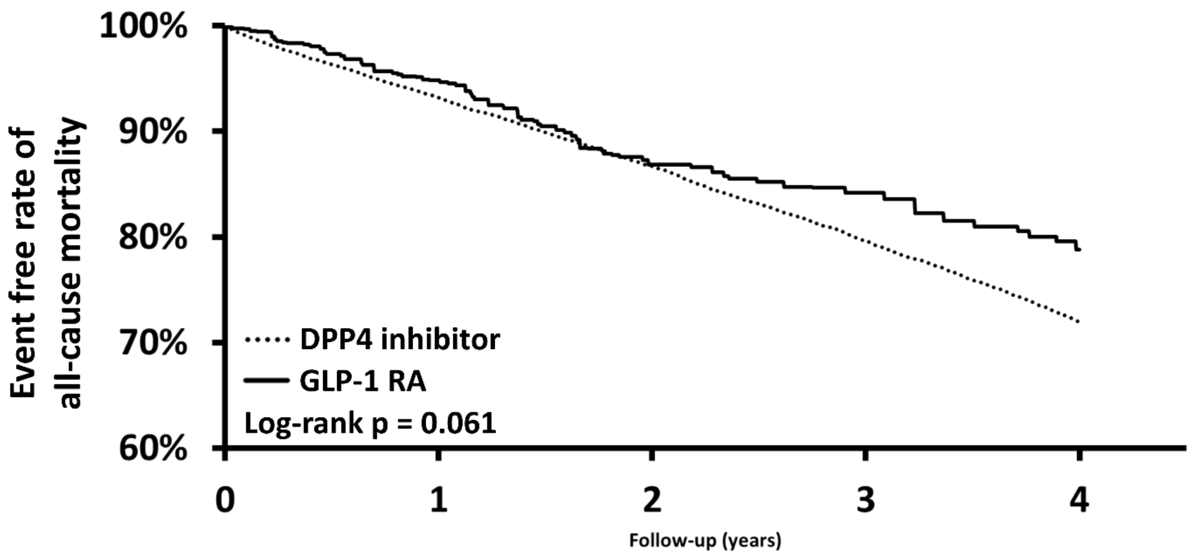

Number at risk					
DPP4 inhibitor	26568	23457	19736	15666	11492
GLP-1 RA	603	461	265	197	143

© 2022 Chen JJ et al. *JAMA Network Open*.

eFigure 1B. Kaplan-Meier survival curves of sepsis/infection-related mortality during follow-up after propensity score weighting

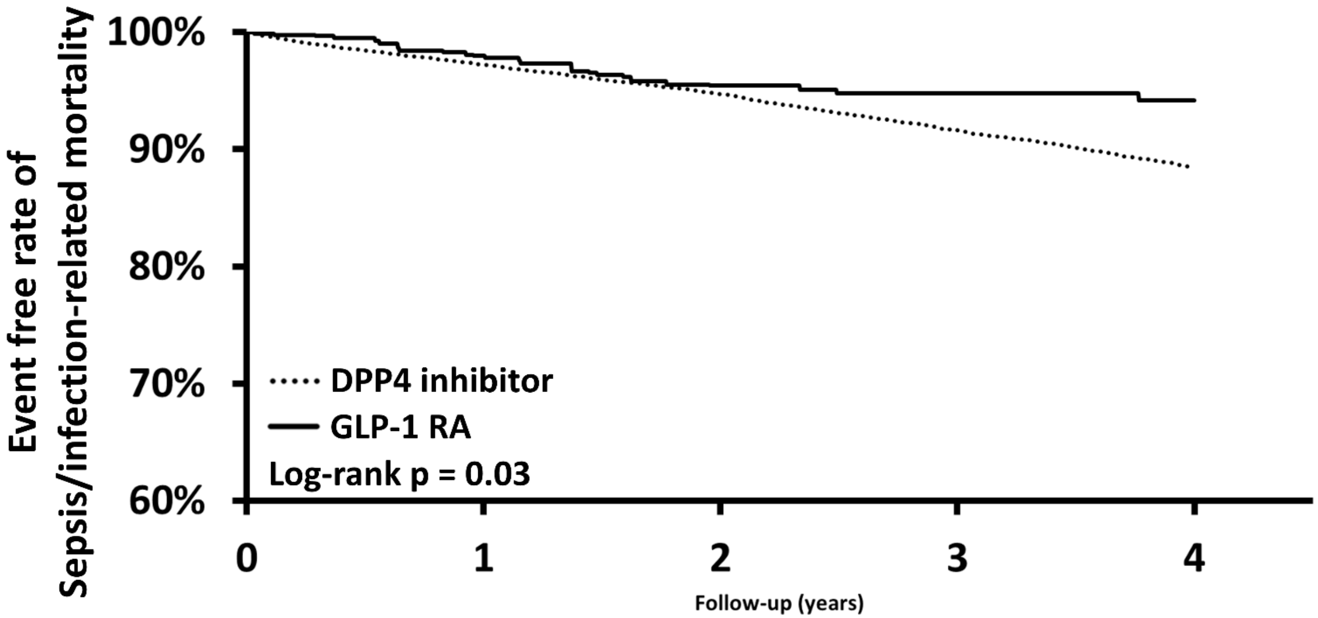

Number at risk					
DPP4 inhibitor	26568	23457	19736	15666	11492
GLP-1 RA	603	461	265	197	143

© 2022 Chen JJ et al. *JAMA Network Open*.

eFigure 1C. Kaplan-Meier survival curves of MACCE-related mortality during follow-up after propensity score weighting

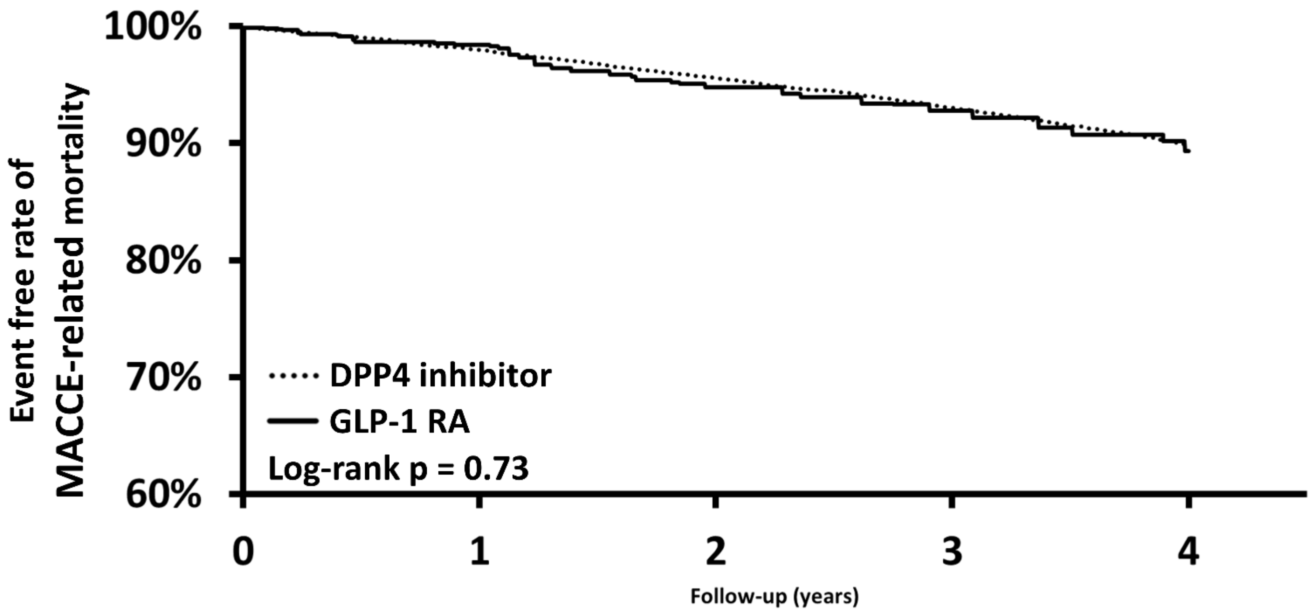

Number at risk					
DPP4 inhibitor	26568	23457	19736	15666	11492
GLP-1 RA	603	461	265	197	143

© 2022 Chen JJ et al. *JAMA Network Open*.

eFigure 2A. Forest plot of subgroup analysis for primary and secondary outcomes. (A) All-cause mortality; (B) MACCE-related mortality; (C) sepsis/infection-related mortality

Independent variables	Incidence rate (per 100 PYs) GLP-1 RA / DPP4 inhibitor	Event of all-cause mortality	GLP-1 RA VS. DPP4 inhibitor HR (95%CI); p value	Interaction p value
**Age**				0.99
< 65	4.03/4.57		0.91(0.66-1.26); 0.58	
65 +	9.31/11.54		0.83(0.60-1.16); 0.27	
**Gender**				0.43
Female	6.11/8.03		0.78(0.57-1.06); 0.11	
Male	6.37/7.89		0.83(0.60-1.15); 0.25	
**Coronary artery disease / ischemic heart disease**				0.41
No	5.13/7.14		0.74(0.56-0.98); 0.03	
Yes	6.98/11.32		0.62(0.38-1.01); 0.06	
**Cerebrovascular disease**				0.04
No	6.45/7.45		0.89(0.71-1.12); 0.32	
Yes	3.77/11.73		0.33(0.12-0.86); 0.02	
**ACEi / ARB**				0.66
No	8.15/10.16		0.80(0.55-1.17); 0.25	
Yes	5.58/6.98		0.83(0.63-1.10); 0.20	
**Dialysis**				0.97
No	5.15/6.49		0.84(0.64-1.11); 0.22	
Yes	10.07/15.77		0.61(0.39-0.95); 0.03	

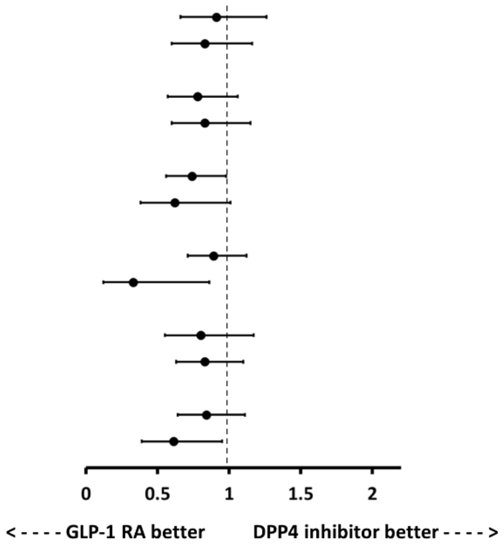

eFigure 2B Forest plot of subgroup analysis for primary and secondary outcomes. (A) All-cause mortality; (B) MACCE-related mortality; (C) sepsis/infection-related mortality

Independent variables	Incidence rate (per 100 PYs) GLP-1 RA / DPP4 inhibitor	Event of MACCE-related mortality	GLP-1 RA VS. DPP4 inhibitor HR (95%CI); p value	Interaction p value
**Age**				0.82
< 65	1.95/1.63		1.26(0.79-2.00); 0.34	
65 +	4.08/3.55		1.20(0.73-1.98); 0.47	
**Gender**				0.96
Female	3.08/2.47		1.29(0.83-2.00); 0.26	
Male	2.68/2.64		1.06(0.65-1.75); 0.81	
**Coronary artery disease / ischemic heart disease**				0.79
No	2.23/2.23		1.05(0.69-1.60); 0.81	
Yes	3.14/3.90		0.82(0.40-1.69); 0.59	
**Cerebrovascular disease**				0.230
No	2.77/2.35		1.22(0.86-1.74); 0.26	
Yes	2.26/4.08		0.57(0.16-2.00); 0.38	
**ACEi / ARB**				0.62
No	3.69/3.13		1.19(0.67-2.10); 0.56	
Yes	2.43/2.31		1.12(0.73-1.70); 0.61	
**Dialysis**				0.35
No	2.51/2.10		1.28(0.86-1.90); 0.23	
Yes	2.59/5.01		0.51(0.21-1.23); 0.13	

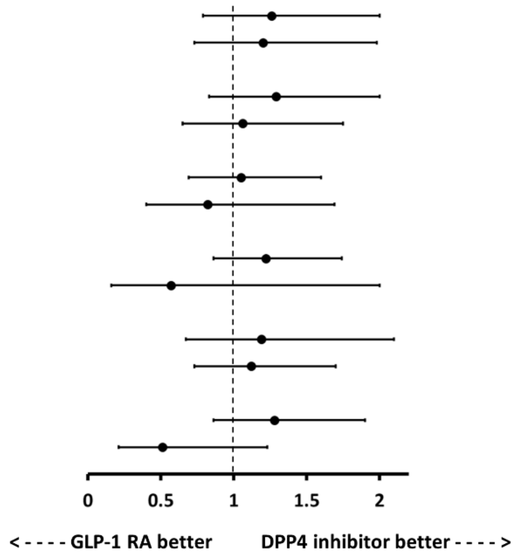

eFigure 2C. Forest plot of subgroup analysis for primary and secondary outcomes. (A) All-cause mortality; (B) MACCE-related mortality; (C) sepsis/infection-related mortality

Independent variables	Incidence rate (per 100 PYs) GLP-1 RA / DPP4 inhibitor	GLP-1 RA VS. DPP4 inhibitor HR (95%CI); p value	Interaction p value
**Age**			0.67
< 65	0.87/1.42	0.63(0.32-1.27); 0.20	
65 +	2.91/4.69	0.64(0.35-1.14); 0.13	
**Gender**			0.10
Female	1.29/3.15	0.41(0.21-0.81); 0.01	
Male	2.01/2.89	0.71(0.40-1.27); 0.25	
**Coronary artery disease / ischemic heart disease**			0.29
No	1.45/2.73	0.55(0.33-0.92); 0.02	
Yes	2.32/4.16	0.56(0.24-1.29); 0.17	
**Cerebrovascular disease**			0.28
No	2.00/2.83	0.72(0.48-1.09); 0.12	
Yes	1.01/4.32	0.23(0.04-1.53); 0.13	
**ACEi / ARB**			0.73
No	2.27/3.98	0.57(0.28-1.17); 0.13	
Yes	1.80/2.58	0.72(0.44-1.17); 0.19	
**Dialysis**			0.52
No	1.40/2.46	0.60(0.35-1.02); 0.06	
Yes	4.60/5.91	0.71(0.37-1.38); 0.31	

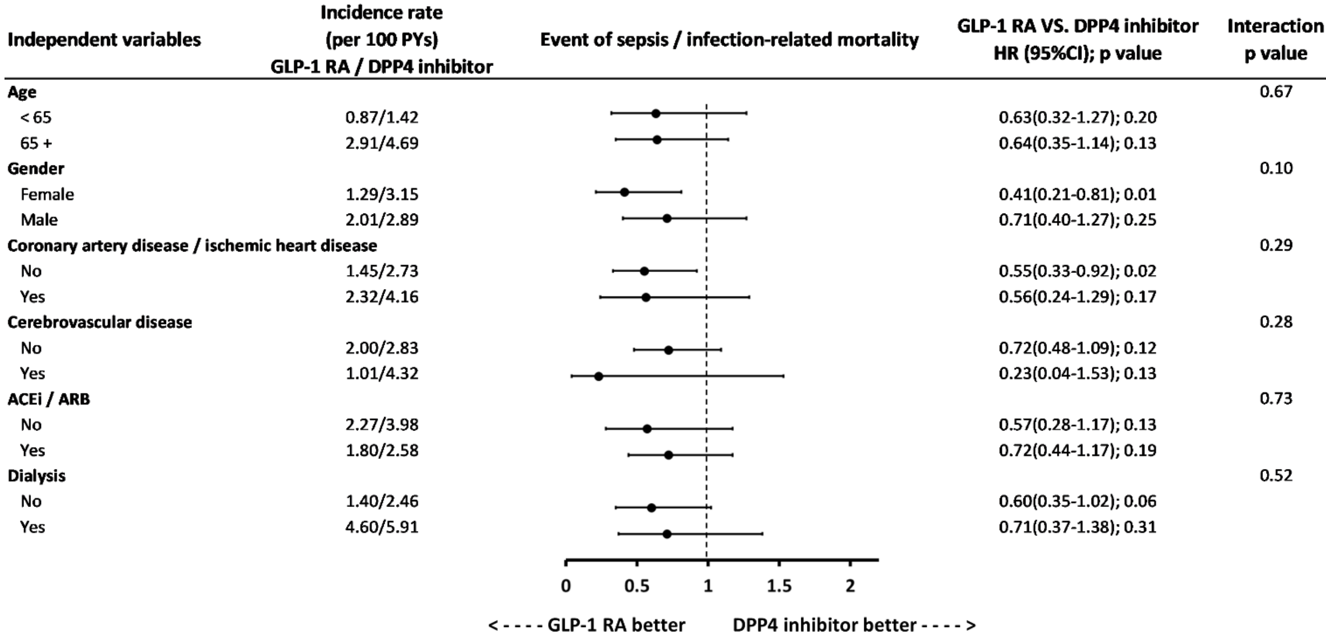

© 2022 Chen JJ et al. *JAMA Network Open*.

eTable 1. ICD9-code and ICD10-code used in this study

Disease	ICD9-code	ICD10-code
Chronic kidney disease (CKD)	016.0, 042, 095.4, 189, 223, 236.9, 250.4, 271.4, 274.1, 403-404, 440.1, 442.1, 446.21, 447.3, 572.4, 580-589, 590-591, 593, 642.1, 646.2, 753, 984	B20, A52.75, C64-68, D30, D41, E11.29, E74.8, M10.30, N20, I70.1, I72.2, M31, I77.3, K76.7, N00-08, N10-15, N18, N28.83, N28.81, N28.1, N28.9, O10.41-43, O12.14, O26.831-839, Q60-4
End-stage renal disease (ESRD)	585	N186
Type 2 DM	250	E11
Kidney transplant	V420	N940
Malignancy	140-208	C
**Comorbidities**		
Hypertension	401-405	I10-I16
Dyslipidemia	272	E78
Liver cirrhosis	571.2, 571.5, 571.6	K70.30-K70.32, K74.5, K74.60, K71.7
Atrial fibrillation	427.31	I48
Peripheral arterial disease (PAD)	440.2, 440.3, 440.8, 440.9, 443, 444.22, 444.8, 444.9	I70.2-I70.9, I73.9
SLE	710	M32
**Infectious disease**		
Sepsis	038, 995.91, 995.92, 020.2, 785.52, 790.7	A40, A41, R65.20, A20.7, R65.21, R78.81
Pneumonia	481-486 (exclude 484)	J13-J18 (exclude J17)
Empyema	510	J86.0, J86.9
Cellulitis	681, 682	L03
Necrotizing fasciitis	728.86	M72.6
Urinary tract infection	590, 595.0, 599.0	N10-N12, N15.1, N15.9, N16, N28.85, N30.00, N30.01, N39.0
Biliary tract infection	576.1, 575.0, 574.00	K83.0, K81.0, K80.00
Brain abscess	324	G06.0, G06.1, G06.2
Liver abscess	572.0	K75.0
Perianal abscess	566	K61
Bacterial meningitis	320	G00
Septic arthritis	711	M00-M03

© 2022 Chen JJ et al. *JAMA Network Open*.

Infection of catheter, device, implant, and graft	996.6, 999.3	T85.7, T80.2
Peritoneal and retroperitoneal infection	567	K65, K67, K68
Osteomyelitis	730.3, 730.8, 730.9	M86.9, M90.8
Infective endocarditis	421	I33
**MACCEs (8 items)**		
Percutaneous Transluminal Coronary (PCI)	33076A, 33076B, 33077A, 33077B, 33078A, 33078B	02103,02104, 02113, 02114, 02123, 02124, 02133, 02134
Coronary artery bypass surgery (CABG)	68023A, 68023B, 68024A, 68024B, 68025A, 68025B	02100, 02110,02120, 02130
Thrombolysis therapy (TT)	B016526248, K000743248, K000744238	3E03317
Cardiogenic shock	785.51,	R57.0
Heart failure (HF)	428	I50
Malignant dysrhythmia	426.0, 426.12-426.13, 426.51-426.52, 426.54, 427.1, 427.4, 427.41-427.42, 427.5	I44.0-3, I45.2, I45.3, I46.9, I47.2, I49.0-3
Myocardial infarction	410	I21, I22
Stroke	430-437	I60-64, G45.0, G45.1, G45.4, G45.8, I67

© 2022 Chen JJ et al. *JAMA Network Open*.

eTable 2. Sensitivity analysis of the primary outcome and secondary outcome after excluding participants with drug switch history

	DPP4 inhibitor (n=26578)		GLP-1 RA (n=588)		GLP-1 RA VS. DPP4 inhibitor
Event type	No. of events / Person-years	Incidence rate (95%CI)	No. of events / Person years	Incidence rate (95%CI)	Hazard ratio (95%CI); p value
Before PSW					
MACCE-related mortality	2009/78127.49	2.57(2.46-2.68)	31/1313.1	2.36(1.53-3.19)	0.95(0.67-1.35); 0.77
Sepsis/infection-related mortality	2366/78127.49	3.03(2.91-3.15)	18/1313.1	1.37(0.74-2.00)	0.46(0.29-0.73); 0.001
All-cause mortality	6252/78127.49	8.00(7.80-8.20)	63/1313.1	4.80(3.61-5.98)	0.61(0.48-0.79); <0.001
After PSW					
MACCE-related mortality	1999/78112.38	2.56(2.45-2.67)	22/1082.84	2.03(1.18-2.87)	0.82(0.54-1.25); 0.35
Sepsis/infection-related mortality	2348/78112.38	3.01(2.88-3.13)	17/1082.84	1.55(0.80-2.29)	0.52(0.32-0.84); 0.008
All-cause mortality	6212/78112.38	7.95(7.76-8.15)	56/1082.84	5.13(3.78-6.48)	0.66(0.51-0.86); 0.002

© 2022 Chen JJ et al. *JAMA Network Open*.

eTable 3. Sensitivity analysis of the primary outcome and secondary outcome after excluding outliers participants with propensity method value which are two times standard deviations from mean

	DPP4 inhibitor (n=25786)		GLP-1 RA (n=557)		GLP-1 RA VS. DPP4 inhibitor
Event type	No. of events / Person-years	Incidence rate (95%CI)	No. of events / Person years	Incidence rate (95%CI)	Hazard ratio (95%CI); p value
Before PSW					
MACCE-related mortality	1983/75861.41	2.61(2.50-2.73)	32/1193.62	2.68(1.75-3.61)	1.07(0.76-1.52); 0.70
Sepsis/infection-related mortality	2342/75861.41	3.09(2.96-3.21)	22/1193.62	1.84(1.07-2.61)	0.61(0.40-0.93); 0.02
All-cause mortality	6177/75861.41	8.14(7.94-8.35)	70/1193.62	5.86(4.49-7.24)	0.74(0.59-0.94); 0.01
After PSW					
MACCE-related mortality	1971/75627.31	2.61(2.49-2.72)	33/1219.99	2.69(1.77-3.61)	1.08(0.76-1.52); 0.68
Sepsis/infection-related mortality	2321/75627.31	3.07(2.94-3.19)	23/1219.99	1.86(1.10-2.63)	0.62(0.41-0.93); 0.02
All-cause mortality	6130/75627.31	8.11(7.90-8.31)	76/1219.99	6.27(4.86-7.67)	0.79(0.63-0.99); 0.04

© 2022 Chen JJ et al. *JAMA Network Open*.

eTable 4. Sensitivity analysis of the primary outcome and secondary outcome after excluding outliers participants with propensity method value which are three times standard deviations from mean

	DPP4 inhibitor (n=26212)		GLP-1 RA (n=612)		GLP-1 RA VS. DPP4 inhibitor
Event type	No. of events / Person-years	Incidence rate (95%CI)	No. of events / Person years	Incidence rate (95%CI)	Hazard ratio (95%CI); p value
Before PSW					
MACCE-related mortality	1998/77098.49	2.59(2.48-2.71)	34/1306.64	2.60(1.73-3.48)	1.05(0.74-1.47); 0.80
Sepsis/infection-related mortality	2357/77098.49	3.06(2.93-3.18)	23/1306.64	1.76(1.04-2.48)	0.59(0.39-0.88); 0.01
All-cause mortality	6222/77098.49	8.07(7.87-8.27)	75/1306.64	5.74(4.44-7.04)	0.73(0.58-0.92); 0.006
After PSW					
MACCE-related mortality	1986/76939.05	2.58(2.47-2.70)	33/1256.26	2.66(1.76-3.56)	1.07(0.76-1.51); 0.70
Sepsis/infection-related mortality	2337/76939.05	3.04(2.91-3.16)	23/1256.26	1.83(1.08-2.58)	0.61(0.41-0.92); 0.02
All-cause mortality	6178/76939.05	8.03(7.83-8.23)	78/1256.26	6.20(4.82-7.58)	0.79(0.63-0.99); 0.04

© 2022 Chen JJ et al. *JAMA Network Open*.

eTable 5. Randomized Controlled Trials Comparing Clinical Effect Between GLP-1 RA and DDP-4 Inhibitors With Significant Difference

**Outcomes**	**Trials**	**Treatment effect of GLP-1 RA**	**Treatment effect of DDP-4 inhibitors**
HbA1c	1860-LIRA-DPP-4[25]	Change from baseline: -1·50% for 1·8 mg liraglutide<br>Change from baseline: -1·24% for 1·2 mg liraglutide	–0·90% for sitagliptin
	AWARD-5[26]	Change from baseline: -1.10% for dulaglutide 1.5 mg<br>Change from baseline: -0.87% for dulaglutide 0.75 mg	-0.39% for sitagliptin
	DURATION-2[27]	Change from baseline: -1.5% for 2 mg exenatide weekly	-0.9% for sitagliptin
	DURATION-NEO-2[28]	Change from baseline: -1.13% for 2 mg exenatide weekly	-0.75% for sitagliptin
	HARMONY 3[29]	Change from baseline: -0.64% for 30mg albiglutide weekly	-0.28% for sitagliptin
	Li 2014[38]	Change from baseline: -1.5%for 1.2 mg liraglutide	-1.23% for sitagliptin<br>-1.25%for vildagliptin
	Leiter 2014[24]	Change from baseline: -0.83% for 30-50mg albiglutide weekly	-0.52% for sitagliptin
	LIRA-SWITCH[30]	Change from baseline: -1.14%for 1.8mg liraglutide	-0.54% for sitagliptin
	PIONEER 3[31]	Oral semaglutide vs sitagliptin estimated treatment difference: –0.3% for 7mg oral semaglutide	

© 2022 Chen JJ et al. *JAMA Network Open*.

		Oral semaglutide vs sitagliptin estimated treatment difference: –0.5% for 7mg oral semaglutide	
	PIONEER 7[32]	Change from baseline: -1.3% for 3, 7, and 14 mg oral semaglutide	-0.8% for sitagliptin
	Seino 2018[34]	Change from baseline: -1.9% for 0.5mg semaglutide weekly<br>Change from baseline: -2.2% for 1.0mg semaglutide weekly	-0.7% for sitagliptin
	SUSTAIN 2[33]	Change from baseline: -1.3% for 0.5mg semaglutide weekly<br>Change from baseline: -1.6% for 1.0mg semaglutide weekly	-0.5% for sitagliptin
	Suzuki 2014[43]	Change from baseline: -2.4% for 0.9mg liraglutide	-1.1% for sitagliptin
	T-Emerge 4[36]	Change from baseline: -1.23% for 10mg taspoglutide weekly<br>Change from baseline: -1.30% for 20mg taspoglutide weekly	-0.89% for sitagliptin
	Zang 2016[37]	Change from baseline: -1.65%for 1.8 mg liraglutide	-0.98% for sitagliptin
Body weight	1860-LIRA-DPP-4[25]	Change from baseline: –3·38 kg for 1·8 mg liraglutide<br>Change from baseline: –2·86 kg for 1·2 mg liraglutide	–0·96 kg for sitagliptin
	AWARD-5[26]	Change from baseline: -3.03 kg for dulaglutide 1.5 mg<br>Change from baseline: -2.60 kg for dulaglutide 0.75 mg	-1.53 kg for sitagliptin

© 2022 Chen JJ et al. *JAMA Network Open*.

	Charbonnel 2013[35]	Change from baseline:-2.8 kg for liraglutide 0.6-1.2 mg	-0.4 kg for sitagliptin
	DURATION-2[27]	Change from baseline: -2.3 kg for 2 mg exenatide weekly	-0.8kg for sitagliptin
	Li 2014[38]	Change from baseline: -6.0 kg for 1.2 mg liraglutide	-0.9kg for sitagliptin<br>-0.8kg for vildagliptin
	Leiter 2014[24]	Change from baseline: -0.79kg for 30-50mg albiglutide weekly	-0.19kg for sitagliptin
	LIRA-SWITCH[30]	Change from baseline: -3.31kg for 1.8mg liraglutide	-1.64kg for sitagliptin
	PIONEER 3[31]	Oral semaglutide vs sitagliptin estimated treatment difference: –1.6kg for 7mg oral semaglutide<br>Oral semaglutide vs sitagliptin estimated treatment difference: –2.5% for 7mg oral semaglutide	
	PIONEER 7[32]	Change from baseline: -2.6kg for 3, 7, and 14 mg oral semaglutide	-0.7kg for sitagliptin
	Seino 2018[34]	Change from baseline: -2.2kg for 0.5mg semaglutide weekly<br>Change from baseline: -3.9kg for 1.0mg semaglutide weekly	-0.0kg for sitagliptin
	SUSTAIN 2[33]	Change from baseline: -4.3kg for 0.5mg semaglutide weekly<br>Change from baseline: -6.1kg for 1.0mg semaglutide weekly	-1.9kg for sitagliptin

© 2022 Chen JJ et al. *JAMA Network Open*.

	Suzuki 2014[43]	Change from baseline: -3.01kg for 0.9mg liraglutide	-1.5kg for sitagliptin
	T-Emerge 4[36]	Change from baseline: -1.8kg for 10mg taspoglutide weekly<br>Change from baseline: -2.6kg for 20mg taspoglutide weekly	-0.9kg for sitagliptin
	Van Gaal 2014[39]	Change from baseline: -2.51kg for 20μg lixisenatide daily	-1.17kg for sitagliptin
	Yan 2019[40]	Change from baseline: -3.6kg for 1.8mg liraglutide	-1.7kg for sitagliptin
	Zang 2016[37]	Change from baseline: -3.17 kg for 1.8 mg liraglutide	-1.08kg for sitagliptin
Beta-cell function	1860-LIRA-DPP-4[25]	HOMA-B change from baseline: 28·70% for 1·8 mg liraglutide<br>HOMA-B change from baseline: 27·23% for 1·2 mg liraglutide	4·18% for sitagliptin
	AWARD-5[26]	HOMA-B change from baseline: 33.6% for dulaglutide 1.5 mg<br>HOMA-B change from baseline: 22.3% for dulaglutide 0.75 mg	6.7% for sitagliptin
	DURATION-2[27]	HOMA-B Ratio of week 28 to baseline: 1.5 for 2 mg exenatide weekly	1.1 for sitagliptin
	SUSTAIN 2[33]	HOMA-B for semaglutide 1.0 mg v.s. sitagliptin: significant improvement	
	T-Emerge 4[36]	HOMA-B change from baseline: 23.5% for 10mg taspoglutide weekly<br>HOMA-B change from baseline: 32.1% for 20mg taspoglutide weekly	10.3% for sitagliptin

© 2022 Chen JJ et al. *JAMA Network Open*.

Lipid profile	AWARD-5[26]	LDL change from baseline: -0.06 mmol/L for dulaglutide 1.5mg<br>LDL change from baseline: 0.02 mmol/L for dulaglutide 0.75 mg	0.12 mmol/L for sitagliptin
	Hiramatsu 2018[42]	LDL change from baseline: significant lower in liraglutide 0.9 mg group	
	SUSTAIN 2[33]	semaglutide 1.0 mg v.s. sitagliptin : increased HDL, decreased triglycerides, decreased VLDL cholesterol	
	Suzuki 2014[43]	LDL change from baseline: -20 mg/dL for 0.9mg liraglutide	0.7 mg/dL for sitagliptin
Blood pressure	Charbonnel 2013[35]	SBP reduction from baseline: –1.9 mm Hg for liraglutide 0.6-1.2 mg	0.9mm Hg for sitagliptin
	DURATION-2[27]	SBP change from baseline, exenatide v.s. sitagliptin : –4 mmHg, 95% CI: –6 to –1	
	Seino 2018[34]	SBP change from baseline, semaglutide 1.0 mg v.s. sitagliptin: –6.01 mmHg, 95% CI – 9.16 to –2.85	
	SUSTAIN 2[33]	SBP change from baseline: –5.1 mm Hg for 0.5mg semaglutide weekly<br>SBP change from baseline: –5.6 mm Hg for 0.5mg semaglutide weekly	-2.3mm Hg for sitagliptin
	Suzuki 2014[43]	SBP change from baseline: –8.5 mm Hg for 0.9mg liraglutide	-1.6mm Hg for sitagliptin

© 2022 Chen JJ et al. *JAMA Network Open*.

Albuminuria	DURATION-2[27]	Exenatide once weekly associated with significantly reduced UACR	
	Hiramatsu 2018[42]	Albuminuria change from baseline: significant lower in liraglutide 0.9 mg group	
	Suzuki 2014[43]	UACR change from baseline: -9.5µg/g for 0.9mg liraglutide	0.7µg/g for sitagliptin

Abbreviations: DDP-4, dipeptidyl peptidase-4; GLP-1 RA, glucagon-like peptide-1 receptor agonist; HOMA-B, homoeostasis model assessment of β-cell function; LDL, low-density lipoprotein; SBP, systolic blood pressure; UACR, urine albumin to creatinine ratio; VLDL, very low-density lipoprotein.

© 2022 Chen JJ et al. *JAMA Network Open*.
